# *In-vivo* topical mucosal delivery of a fluorescent deoxy-glucose delineates neoplasia from normal in a preclinical model of oral epithelial neoplasia

**DOI:** 10.1038/s41598-018-28014-8

**Published:** 2018-06-27

**Authors:** Rahul Pal, Paula Villarreal, Suimin Qiu, Gracie Vargas

**Affiliations:** 1 Center for Biomedical Engineering, The University of Texas Medical Branch, Galveston, Tx 77555 USA; 2 Department of Pathology, The University of Texas Medical Branch, Galveston, Tx 77555 USA; 3 Center for Cancers of the Head and Neck, The University of Texas Medical Branch, Galveston, Tx 77555 USA; 4 Department of Neuroscience and Cell Biology, The University of Texas Medical Branch, Galveston, Tx 77555 USA

## Abstract

Metabolic imaging of oral cavity mucosal surfaces could benefit early detection of oral squamous cell carcinoma (OSCC) and oral epithelial dysplasia (OED). Fluorescent deoxy-glucose agents provide contrast for glucose metabolism similar to ^18^FDG-PET imaging and allow use of optical imaging, which provides high resolution and lower potential cost. However, *in-vivo* topical mucosal delivery of fluorescent deoxy-glucose agents without injection or tissue resection has not been shown. We introduce *in-vivo* optical imaging of neoplasia following mucosal delivery of 2-deoxy-2-[(7-nitro-2,1,3-benzoxadiazol-4-yl)amino]-D-glucose (2-NBDG) in an OSCC/OED hamster model and demonstrate uptake into epithelium across the mucosal surface without injection or disrupting the epithelium. 2-NBDG fluorescence intensity following 30-minutes topical application was 6-fold and 4-fold higher in OSCC and OED, respectively, compared to normal mucosa. Receiver operator characteristic analysis show 83% sensitivity and 73% specificity for detection of neoplasia vs benign (normal and inflammation). Faster 2-NBDG fluorescence temporal decay in neoplasia indicated higher uptake and glucose metabolic rate than normal mucosa. Mucosal delivery of 2-NBDG by topical application to the *in-vivo* oral surface is feasible and delineates neoplasia from normal mucosa, providing *in-vivo* noninvasive molecular imaging of dysregulated glucose metabolism, which could benefit preclinical studies of carcinogenesis or be developed for use in early detection.

## Introduction

Incidence of Oral Squamous Cell Carcinoma (OSCC) (~90% of oral cancers), has increased over the past two decades with 640,000 new cases per year worldwide^1^. The OSCC five-year survival rate largely depends on the stage at diagnosis and may be as low as 32% when cancer has spread to distant sites, but can be as high as 80% when detected locally^2^. Transformation of OSCC involves pre-cancerous phases including oral epithelial dysplasia (OED). OED, particularly moderate and severe, is associated with a high risk of progression to invasive cancer^3^. Although OSCC is considered preventable if caught early, its generally late stage diagnosis results in substantial global impact causing 145,353 deaths worldwide in 2012^4^. Clinical detection of OSCC and OED is by conventional oral examination (COE) and surveillance with subsequent biopsy. Histopathological grading of suspected lesions is the current gold standard for diagnosis. COE to identify potential OSCC/OEDs is typically by white light examination and palpation of the oral cavity with results highly dependent on experience of the examiner^5,6^. Neoplastic lesions are often diffuse and heterogeneous and, because of the lack of contrast relative to surrounding areas, may go undetected by COE^7^. Therefore, OSCC is often detected at late stages, making efficient detection of oral neoplasia, including high-risk precancers (OEDs), with improved contrast and accurate assessment of neoplastic potential, critical^8^.

Improved identification of neoplastic areas could be achieved using a molecular contrast agent that exploits characteristics of neoplastic epithelium, such as increased glucose metabolism, revealed in tumor masses by clinical imaging (^18^FDG-PET). Neoplastic tissues have a greater glucose demand than healthy tissues^9^ with the rate of glucose consumption increasing to meet the energy demand of highly proliferating neoplastic cells that favor aerobic glycolysis, a phenomenon known as the Warburg Effect. This response includes over-expression of glycolytic enzymes and glucose transporters (GluTs)^10^ and forms the basis for monitoring glucose consumption by ^18^FDG-PET for clinical detection of tumors. However, in OSCC, ^18^FDG-PET is primarily used when planning adjuvant treatment and, due to resolution limits (~4 mm)^11^, is not optimal for detection of precancers confined to the epithelium (hundreds of microns thick), or of early cancers on the oral cavity mucosal surface. Thus, optical contrast agents for glycolysis could provide a better approach for oral OED and early OSCC detection, as optical imaging provides micron-level resolution and can image over several millimeters-to-centimeters across the surface. Fluorescent deoxy-glucose probes, such as 2-(N-(-7-Nitrobenz-2-oxa-1,3-diazol-4-yl)Amino)-2-Deoxyglucose (2-NBDG), an optical equivalent of ^18^FDG, have been shown to preferentially accumulate in tumors in a process similar to ^18^FDG.

While fluorescent deoxy-glucose probes (primarily 2-NBDG) have been investigated for potential optical contrast in preclinical studies of oral neoplasia^12–17^, previous delivery methods were systemic injection of the probe or immersion of *ex-vivo* resected tumors or biopsies in a probe solution. The latter immersion methods have been described as topical, but delivery across intact epithelium is not possible with resected tissue immersed in a solution. *In-vivo* topical mucosal delivery of 2-NBDG would provide several advantages including selective application of the agent to the imaging site, reduced fluctuations in drug levels that can occur through systemic injection, and avoidance/reduction of potential systemic reactions or toxicity. With these advantages, plus easy application, *in-vivo* topical mucosal delivery of 2-NBDG on the oral cavity could facilitate *in-vivo* preclinical studies requiring metabolic monitoring of the mucosal surface and lead to potential clinical use of 2-NBDG and similar agents to aid OED and early OSCC detection.

Here, we report on a new approach for full *in-vivo* optical metabolic imaging of oral epithelial neoplasia, which is based on topical mucosal delivery of 2-NBDG, on the fully intact mucosal surface. We evaluated *in-vivo* topical mucosal delivery of 2-NBDG for delineation of neoplasia in a preclinical model for OED and OSCC using widefield fluorescence (WF) imaging and show: (1) successful *in-vivo* uptake of 2-NBDG in normal, inflamed, and neoplastic mucosa and (2) enhanced uptake and utilization in neoplasia (OED and OSCC) compared to normal or inflamed tissue (common confounder in oral neoplasia detection). *In-vivo* topical application of 2-NBDG could open new avenues of WF imaging to detect tissue areas with altered glucose uptake and metabolism associated with neoplasia in preclinical models and, ultimately, in humans.

## Methods

### Animal Model

Animal studies were approved by the Institutional Animal Care and Use Committee of the University of Texas Medical Branch and conformed to the Guide for the Care and Use of Laboratory Animals. Four-week old male Golden Syrian Hamsters (Harlan Laboratories, Indianapolis) were used. OSCC/OED were induced by topical treatment of 0.5% 9,10-dimethyl-1,2-benzanthracene (DMBA) in mineral oil on the left buccal (cheek) pouch of hamsters, three times per week for 8–12 weeks^18^. Sham control animals were treated with mineral oil following the same topical application and time course. This model has been used in numerous preclinical studies and demonstrates histological and molecular similarities to human OED and OSCC^18,19^. In an additional group, a Sodium Lauryl Sulfate (SLS) induced inflammation model was used. Hamsters were treated topically with a solution of 1.4% SLS, 29% calcium pyrophosphate and 18% glycerol in sterilized water^20,21^, for four consecutive days and used for imaging studies on the 5th day^20,22,23^. SLS treatment results in a generalized inflammatory response with early neutrophil and mononuclear cell infiltration and over several days also results in hyperkeratinization, acanthosis, and benign epithelial hyperplasia. A temporal study with three DMBA-treated and two control hamsters was performed that provided 8 OSCC, 7 OED (mild, moderate, severe) and 8 normal sites. In a second group of single time-point study, Sixteen DMBA-treated, four SLS-treated and eight control animals were used providing a total of 23 OSCC, 35 OED (mild, moderate, severe), 19 inflamed and 26 normal sites. Immediately before imaging, animals were anesthetized with intraperitoneal injection of 100-mg/kg ketamine and 2.5-mg/kg xylazine.

### WF Imaging

**Figure 1:**
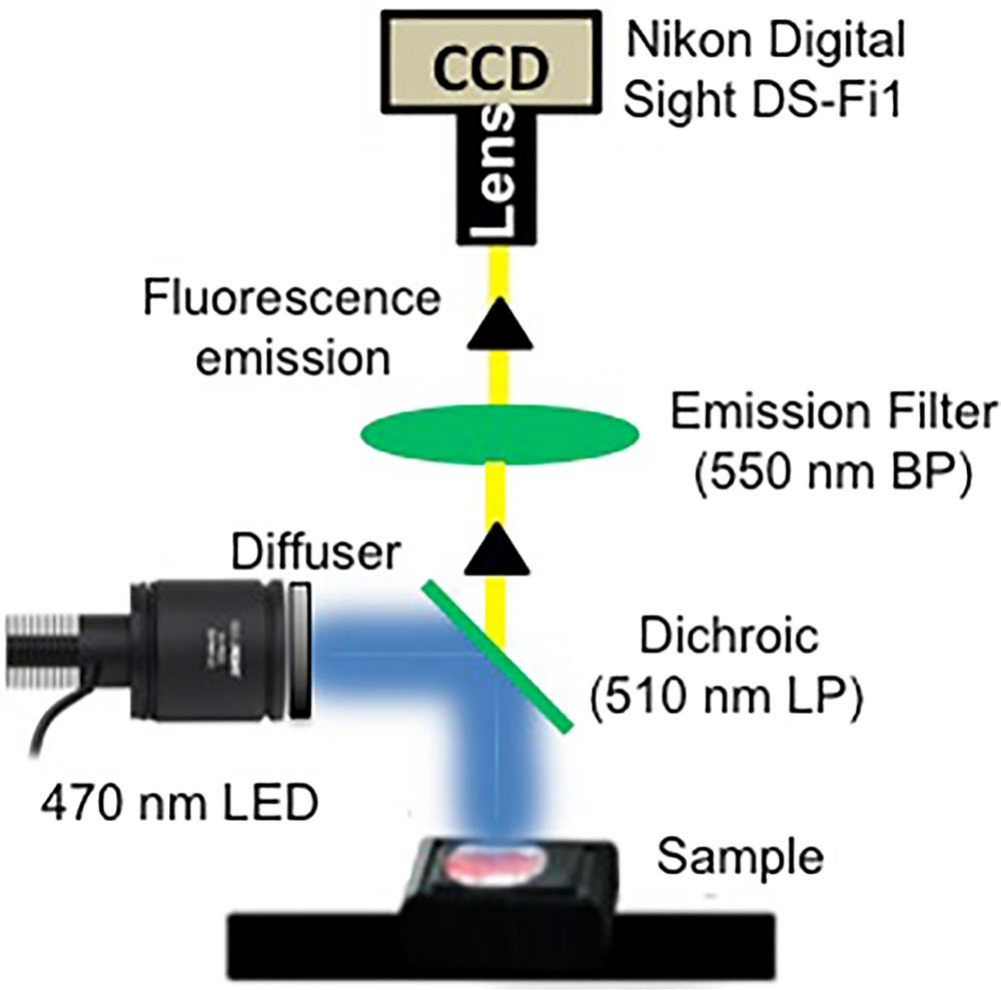
WF imaging setup. A high power 470 nm LED as excitation source, 510 nm LP dichroic and a 550 nm BP filter were used to collect fluorescence into a Nikon digital camera with a Bayer mask.

The buccal pouch was gently pulled out of the oral cavity, stretched onto a flat sample holder, secured in place using pins and rinsed with PBS. A reference photographic image similar to COE using white light illumination of the buccal pouch and a baseline fluorescence image prior to topical application of 2-NBDG were taken using a custom built WF imaging system. The WF system (Fig. 1) consisted of a collimated 470 nm LED (M470L3-C1, ThorLabs, NJ) excitation light source, a diffuser, a long pass 510 nm dichroic (Edmund Optics, NJ) filter to direct excitation light onto the sample from above and to transmit remitted fluorescence, and a 550 nm (45 nm BP) (Edmund Optics, NJ) emission filter (OD >6 beyond passband) above the dichroic. Fluorescence detection was through AF Micro NIKKOR 60 mm 1:2.8 lens attached to a scientific camera, Nikon DS Fi1 (Nikon, Japan), with a 5-megapixel CCD sensor that captured images with 2560 × 1920 pixels and 8-bits per channel. Illumination diameter at the sample was 5 cm and image field-of-view (FOV) was 4 × 3 cm. The diffuser ensured an even illumination across the 4 cm diameter FOV with less than 5% loss in power density at the edges beyond the FOV. Resolution of the system was measured using a U.S. Air Force (USAF) spatial resolution target in which the smallest line spacing clearly discriminated was 24.8 µm (group 4, element 3). System alignment and calibration was performed before every imaging session using fluorescence standard slides (Edmund Optics, NJ). All imaging was performed with f-number of 2.8, with exposure times 200–800 ms. A standard black metal sample holder served as a background reference and was imaged at the beginning of every imaging session to capture and subtract reference background from fluorescence images.

### 2-NBDG topical application

1 ml 2-NBDG (1 mg/ml) in sterile PBS (pH 7.4) was applied on right buccal mucosa for 30-minutes at room temperature in dark. Care was taken to cover the entire surface with 2-NBDG. After topical exposure tissues were rinsed for 2 minutes with PBS and excess 2-NBDG was wiped from the tissue surface using sterile gauze. Tissue was rinsed with PBS for 1 minute prior to WF imaging.

#### Measurement of 2-NBDG fluorescence decay

The first imaging time point after the 30-min topical delivery was at 5 minutes due to the time required to rinse the tissue and reposition the animal under the imaging system. Imaging was continued at 5-minute intervals for 120 minutes post 2-NBDG topical application. Camera exposure and gain for individual experiments stayed same at all time points.

### Differential 2-NBDG uptake across pathological groups

The single time-point set of hamsters were imaged at the 60-minute post 2-NBDG application. To evaluate 2-NBDG uptake across neoplastic and normal regions, regions of interests (ROIs) for intensity analysis and biopsy were selected across all areas of the buccal pouch. Selection criteria for ROIs included: (1) Physical appearance under white light examination: areas with visible surface abnormalities in texture, color and swelling were included as potentially neoplastic while areas with no abnormal surface appearance were selected as potentially normal (final classification was determined by histopathology); (2) At least 5 mm away from tissue edges including areas where tissue was secured on the sample holder; (3) regions away from visible raised areas/folds (possible surface accumulation of fluorophores in folded tissue); (4) away from necrotic tumors and (5) areas void of visible specular reflectance which could not be eliminated from all areas completely despite filtering for fluorescence (such areas comprised less than ~5% of the pouch). Biopsies were obtained using a 2-mm diameter punch biopsy from each identified ROI and processed for histopathology.

#### Histology and Immunohistochemistry

Biopsied tissues were fixed in 10% formalin, embedded in paraffin, sectioned (10 µm thickness), and stained with hematoxylin and eosin (H&E) or processed for immunohistochemistry (IHC). An experienced pathologist using standard World Health Organization (WHO) criteria based on nuclear and cytologic atypia, integrity of basement membrane and epithelial cell proliferation performed histopathological grading. For IHC, 10 µm thick paraffin embedded tissue sections were dewaxed in xylene and rehydrated in decreasing concentration of alcohol. Antigen retrieval was performed with Sodium Citrate (pH 6.0) at sub boiling temperature for 20 minutes. Glucose transporter-1 (GluT1) primary antibody (Lifespan Biosciences, WA) was used at 1:200 dilution and pyruvate dehydrogenase kinase (PDK) primary antibody (Abcam, MA) was used at 1:400 dilution. Secondary antibody was applied for 30 minutes at 37 °C and slides were developed with DAB (Vector Laboratories, CA) and counterstained with Harris Hematoxylin.

### Validation of 2-NBDG uptake by epithelial cells in the intact mucosa

Confirmation of 2-NBDG uptake was obtained by *in-vivo* and *ex-vivo* tissue imaging with and without topically applied 2-NBDG. Mucosal tissue was imaged *in-vivo* using a two-photon microscope (Prairie Technologies Ultima IV, Middleton, WI) before and after 2-NBDG application. 800 nm excitation, 500–600 nm emission filter and 28 mW power was used for imaging through a 25×, 1.2 N.A. objective. Images were taken from superficial and basal cell layers. In other assessments, *ex-vivo* tissue biopsies (2.5 mm diameter) immediately after 30 min topical application of 2-NBDG (or saline) were imaged to identify spectral characteristics of the fluorescence signal using a confocal microscope having a spectral detection unit (FV1000, Olympus, Japan). 488 nm excitation and a 20×, 0.75 N.A. objective was used. Fluorescence emission was collected between 510 nm–670 nm with one image acquired at every 5 nm band-width. Laser power remained unchanged between 2-NBDG and saline groups to allow for intensity comparisons. In a third assessment, biopsied tissues were embedded in OCT and flash frozen in liquid nitrogen. Then, tissues were cryosectioned (10 µm thickness) immediately and imaged in cross-section using an Olympus IX71 inverted fluorescence microscope (Olympus, Center Valey, PA). 488 nm excitation, BP 550/45 nm emission and a 10×, 0.3 N.A. objective was used.

### Image Analysis

WF images in the green channel containing 2-NBDG fluorescence were background subtracted, enhanced for brightness/contrast and sites on the buccal pouch were analyzed for fluorescence intensity with selection criteria described above. For each selected site (R1) three small regions (r1, r2, r3) were chosen and average intensities were calculated (I_r1,ave_; I_r2,ave_; I_r3,ave_) from the baseline image and 60-min post 2-NBDG image using ImageJ (NIH, Version 1.48 u). Average intensity of R1 was then expressed as (I_R1_ = [I_r1,ave_ + I_r2,ave_ + I_r3,ave_]/3). Increase in 2-NBDG fluorescence over baseline (ΔF_2-NBDG_) was calculated as I_R1,Post 2-NBDG_/I_R1,Pre 2NBDG_. ΔF_2-NBDG_ was statistically compared between the groups (normal, OED, inflammation, OSCC) by single factor ANOVA and Tukey’s post hoc analysis. Receiver operator characteristic (ROC) curves were created using SPSS (IBM, NY). HIstopathological grading was used as the gold standard to generate ROC curves and obtain ΔF_2-NBDG_ threshold values, which were used to calculate area under the curve (AUC), sensitivity and specificity.

2-NBDG fluorescence decay rate over a 2-hour period was calculated using an approximation used in previous studies^24^. For each site (R1) five small ROIs (r1, r2, r3, r4, r5) were chosen and average intensities were calculated at each time point. For each ROI the rate of 2-NBDG fluorescence decay was approximated as (I_max_ − I_120_)/(T_120_ − T_max_) where I_max_ and T_max_ indicate maximum 2-NBDG fluorescence and time of maximum 2-NBDG fluorescence respectively which was typically 10-min post removal of 2-NBDG. This approximation allowed for a comparison of 2-NBDG decay times between groups despite the potential complexity in modeling fluorescence response that could depend on simultaneous uptake, multiple-step glycolysis during utilization and tissue properties.

## Results

### 2-NBDG uptake in mucosal tissue after topical application

**Figure 2:**
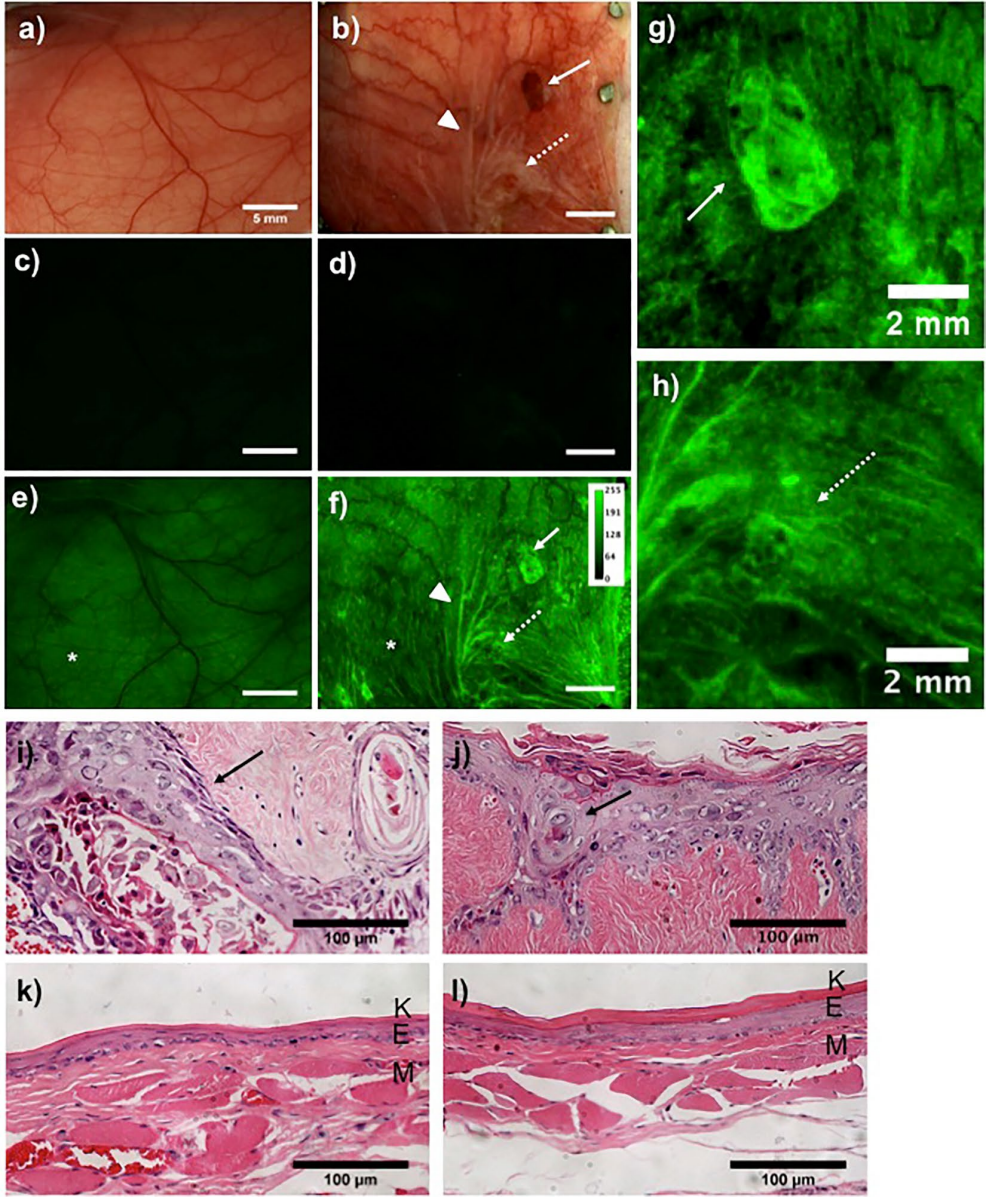
2-NBDG uptake in mucosal tissue after topical application. White light images of normal (**a**) and DMBA treated (**b**) cheek pouch mucosa; Pre 2-NBDG baseline fluorescence images of the normal (**c**) and DMBA treated (**d**) cheek pouch mucosa; Post 2-NBDG fluorescence of the normal (**e**) and DMBA treated cheek pouch mucosa (**f**) are shown. OSCC (solid arrow) and OED (dotted arrow) indicated in (**f**) are zoomed in and shown in (**g**) and (**h**) respectively. Arrows in (**a**–**h**) indicate ROIs with solid arrows for OSCC and dotted arrows for OED. Arrow heads in (**b**) shows tissue folds and in (**f**) shows 2-NBDG accumulation in those folds. (**i**–**l**) H&E stained sections of the OSCC site from (**g**), OED site from (**h**), normal site from (**e**, “*”) and normal (**f**, “*”) tissues are shown respectively. “*” in (**e**,**f**) indicate normal epithelium. K: keratinizing layer; E: epithelium; M: extracellular matrix. Arrows in (**i**,**j**) indicate neoplastic epithelial cells. Scale bar is 5 mm unless specified in the image.

Figure 2 shows representative images of control and OSCC hamster buccal mucosa. White light images of normal and DMBA-treated oral mucosa are shown in Fig. 2a,b, respectively. No surface abnormalities in normal samples were visible, while the DMBA treated mucosa showed exophytic tumors (solid arrow) and in some cases, precancerous lesions (dotted arrow) with rough surface texture (hyperkeratinization) and a red or white color. Both normal and DMBA treated mucosa showed low background autofluorescence pre- 2-NBDG application (Fig. 2c,d, respectively). 2-NBDG topical application significantly increased fluorescence intensity in both normal (Fig. 2e) and DMBA (Fig. 2f) groups, implying buccal mucosal uptake of 2-NBDG.

2-NBDG fluorescence intensity in neoplasia was higher than in normal tissue. Normal mucosa showed homogeneous distribution of 2-NBDG fluorescence across the full mucosal surface, while neoplastic tissue showed heterogeneous fluorescence across the mucosa, punctuated with hotspots (Fig. 2f: arrows). 2-NBDG accumulation in tissue folds also created fluorescence hotspots (Fig. 2f “arrow head”) and were excluded from analysis. Figure 2g,h show a tumor (Fig. 2f: solid arrow) and an OED (Fig. 2f: dotted arrow) respectively, which were also visible via white light. These areas showed 2-NBDG fluorescence hotspots and were graded by histopathology as OSCC (Fig. 2i) and OED (Fig. 2j). OSCC histopathology showed loss of layered epithelial structure and complete invasion, while the OED showed increased epithelial thickness and nuclear density without basement membrane disruption. Along with visible neoplastic lesions, additional hotspots were observed (Fig. 2f, dotted circles), that were not visible under white light examination. Histopathologic inspection of these were found to harbor OED as discussed below. Areas with low 2-NBDG fluorescence were also assessed for pathology. Two such regions from normal epithelium (Fig. 2e: “*”) and DMBA treated epithelium (Fig. 2f: “*”) are shown in Fig. 2k and Fig. 2l, respectively. These revealed normal epithelial morphology, distinct thin layers of keratinization, and epithelium and stroma with no visible cytologic abnormalities.

**Figure 3:**
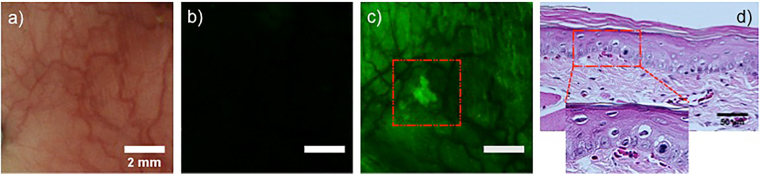
2-NBDG uptake by OEDs not visible in COE. White light (**a**) pre 2-NBDG (**b**) and post 2-NBDG (**c**) images of a moderate dysplasia are shown. The region of high 2-NBDG uptake in (**c**) is outlined. (**d**) H&E stained section of the ROI.

While visible hyperkeratinizing lesions had OED, several OEDs were found in tissues with increased 2-NBDG uptake with no visible surface abnormalities–(in 6 of 35 OED sites sampled). Figure 3 shows an OED lacking gross surface abnormalities. The site appeared normal (Fig. 3a) with low baseline autofluorescence (Fig. 3b). However, 2-NBDG topical application revealed a hotspot (Fig. 3c). Histopathology (Fig. 3d) showed cytologic features indicating moderate OED. This finding is particularly important since abnormal uptake of 2-NBDG revealed an area of moderate OED that was visually unsuspicious.

### Depth-resolved microscopic assessment of 2-NBDG penetration via topical application

**Figure 4:**
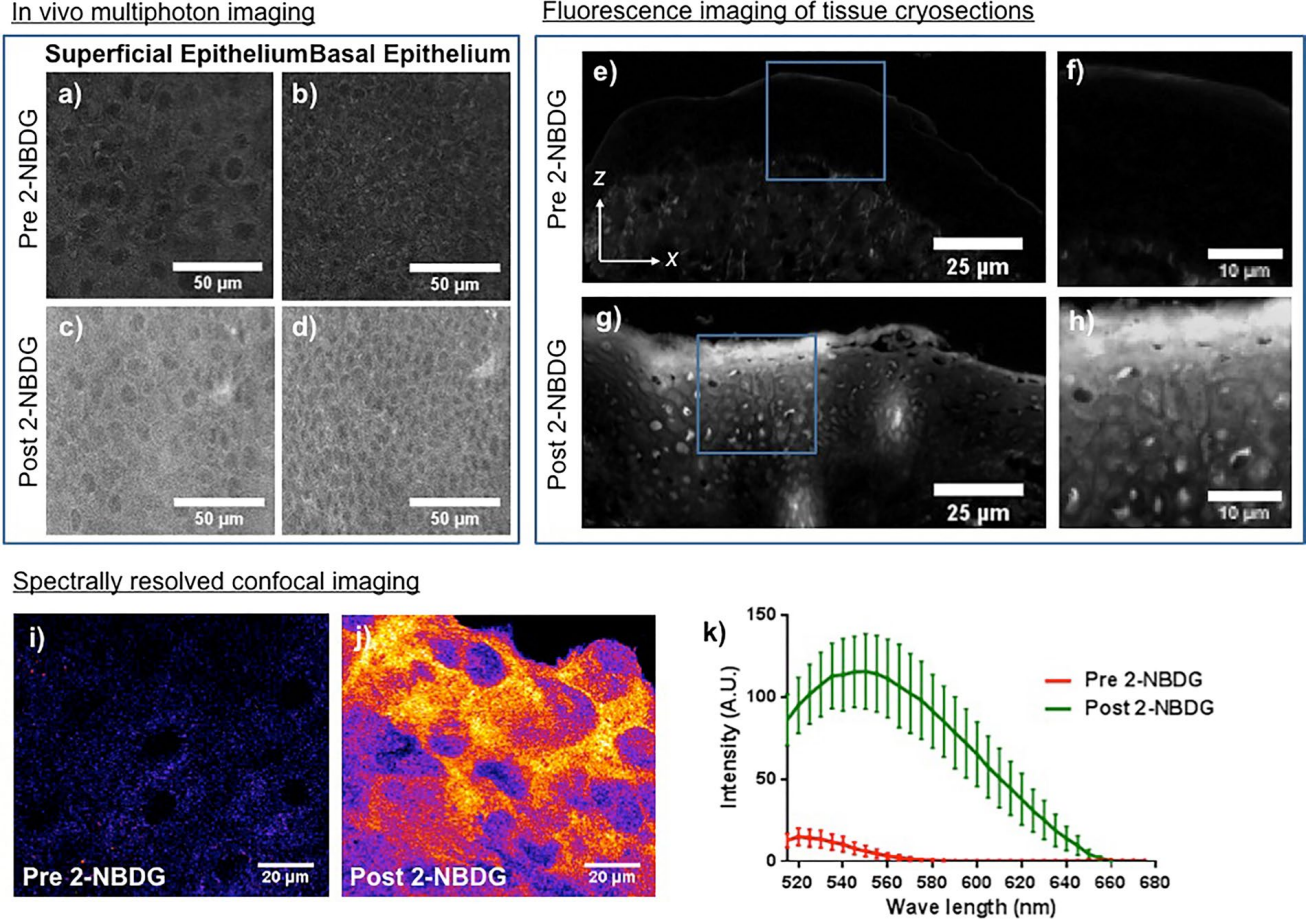
2-NBDG delivery in deep mucosal layers. Images from two-photon microscopy of superficial (**a**) and basal (**b**) epithelium without 2-NBDG are shown. Images were obtained using 800 nm excitation and 500–700 nm bandpass emission filter. (**c**) and (**d**) shows two-photon microscopy images of superficial and basal epithelial cells, respectively, after topical application of 2-NBDG for 30 minutes; (**e**) cryosection of mucosal tissue without 2-NBDG; (**f**) zoomed in image of the epithelium from the area outlined in (**e**); (**g**) cryosection of mucosal tissue with 2-NBDG; (**h**) zoomed in image of the epithelium from the area outlined in (**g**); (**i**) and (**j**) are confocal microscopy images at 550 nm emission before and after 2-NBDG application, respectively; (**k**) Fluorescence emission spectrum of epithelial tissue with (green) and without (red) topically applied 2-NBDG.

Confirmation of 2-NBDG uptake by epithelial cells through nucosal surface only was performed by microscopic evaluation of subsurface epithelial layers. Figure 4a–c show baseline two-photon fluorescence images from superficial and basal epithelium before 2-NBDG application. *In-vivo* topical 2-NBDG for 30-minutes resulted in increased fluorescence in both superficial (Fig. 4b) and basal (Fig. 4d) epithelial layers of the intact mucosa, indicating penetration of 2-NBDG into the basal epithelium. Figure 4e–h show cryosections of biopsies with and without 2-NBDG, in tissue cross-sections imaged using a fluorescence microscope. Tissues without 2-NBDG showed low autofluorescence (Fig. 4e) while those with 2-NBDG showed increased fluorescence (Fig. 4g) in the 2-NBDG excitation/emission channel. Figure 4f,h (zoomed in areas in Fig. 4e,g, respectively), show intracellular localization of 2-NBDG. To spectrally characterize this signal as final validation of epithelial 2-NBDG uptake, biopsies from tissues with and without topical 2-NBDG were imaged using confocal microscopy with spectral capabilities. Fluorescence spectra showed the full 2-NBDG emission spectrum. Figure 4I,j are from the 545–550 nm band pass emission window (2-NBDG emission peak) showing baseline autofluorescence before 2-NBDG application, and fluorescence after topical 2-NBDG, respectively. Epithelial cells in Fig. 4j showed greater fluorescence intensity than in Fig. 4i. The fluorescence emission spectrum obtained from confocal images is shown in Fig. 4k. Tissue without 2-NBDG (‘red’) showed weak autofluorescence emission (520 nm peak), while tissue with topical 2-NBDG (‘green’) showed strong fluorescence emission (550 nm peak), indicating penetration of 2-NBDG into the epithelium and epithelial uptake.

**Figure 5:**
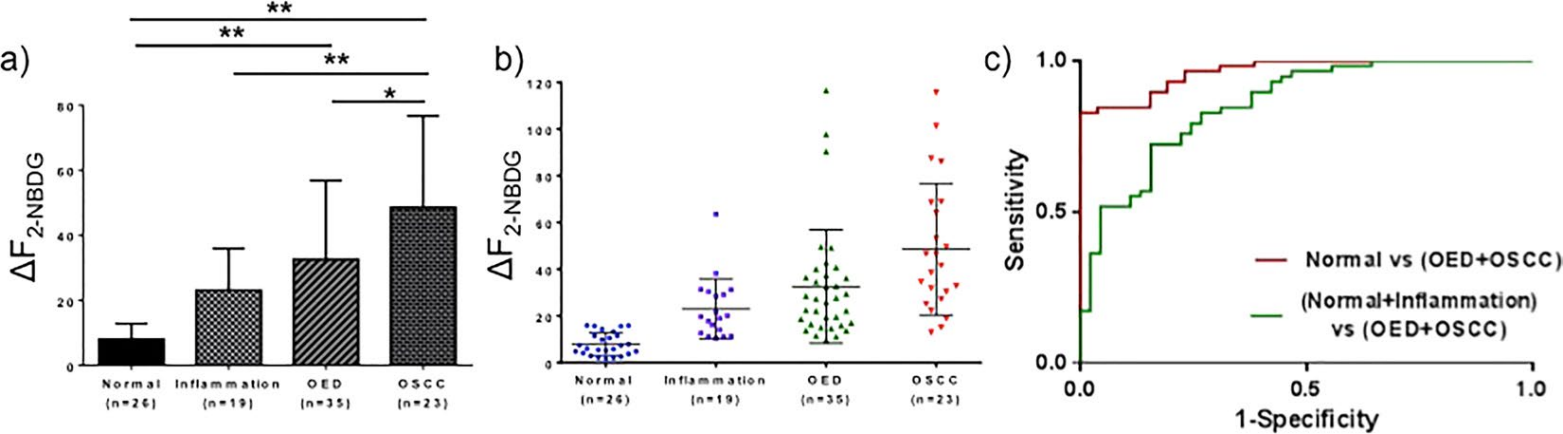
ΔF2-NBDG distribution and ROC curves. (**a**) ΔF_2-NBDG_ as measured from WF imaging for normal, inflammation, OED and OSCC. Mild, moderate and severe dysplasias were grouped into the OED group. (**b**) Scatter plot of 2-NBDG uptake for each sample across pathological groups. Note that inflammation, OED and OSCC show a broader distribution than normal. (**c**) ROC curves using ΔF_2-NBDG_ with or without inflammation in the normal group; green: inflammation grouped with normal (Sensitivity: 83%, Specificity: 73%, AUC: 0.86); red: normal alone compared with OED and OSCC (Sensitivity: 90%, Specificity: 85%, AUC: 0.97). *p < 0.05, **p < 0.01.

Figure 5 shows ΔF_2-NBDG_ across normal, inflammation, OED and OSCC. Mild, moderate, and severe dysplasia were grouped into the OED group. Results show a statistically significant increase in ΔF_2-NBDG_ (Fig. 5a) in OSCC (48.53 ± 28.16) compared to normal (7.99 ± 4.85), inflammation (23.02 ± 12.91) and OED (32.57 ± 24.25). ΔF_2-NBDG_ in the OED group was also significantly higher than normal, but did not differ from the inflammation group. Inflammation showed higher ΔF_2-NBDG_ than normal, but this was not statistically significant. Figure 5b shows fluorescence distribution for individual groups. Distribution for normal was narrow and clustered in low fluorescence values. OED and OSCC revealed a broad range, mostly at higher intensities, respectively. Intensities from inflammation overlapped with both normal and OED. As is seen in the ROC curves, this overlap affected 2-NBDG uptake as a measure of neoplastic abnormality (Fig. 5c). With histopathology as the gold standard and ΔF_2-NBDG_ as a discriminating factor (between normal and neoplasia), ROC analysis showed high sensitivity and specificity (90% and 85% respectively), with AUC of 0.97 when non-inflamed normal tissue was compared with neoplastic (OED and OSCC) tissue. However, inflammation present in the normal group reduced sensitivity and specificity to 83% and 73%, respectively, and the AUC to 0.86.

### Immunohistochemistry of glycolysis markers

**Figure 6:**
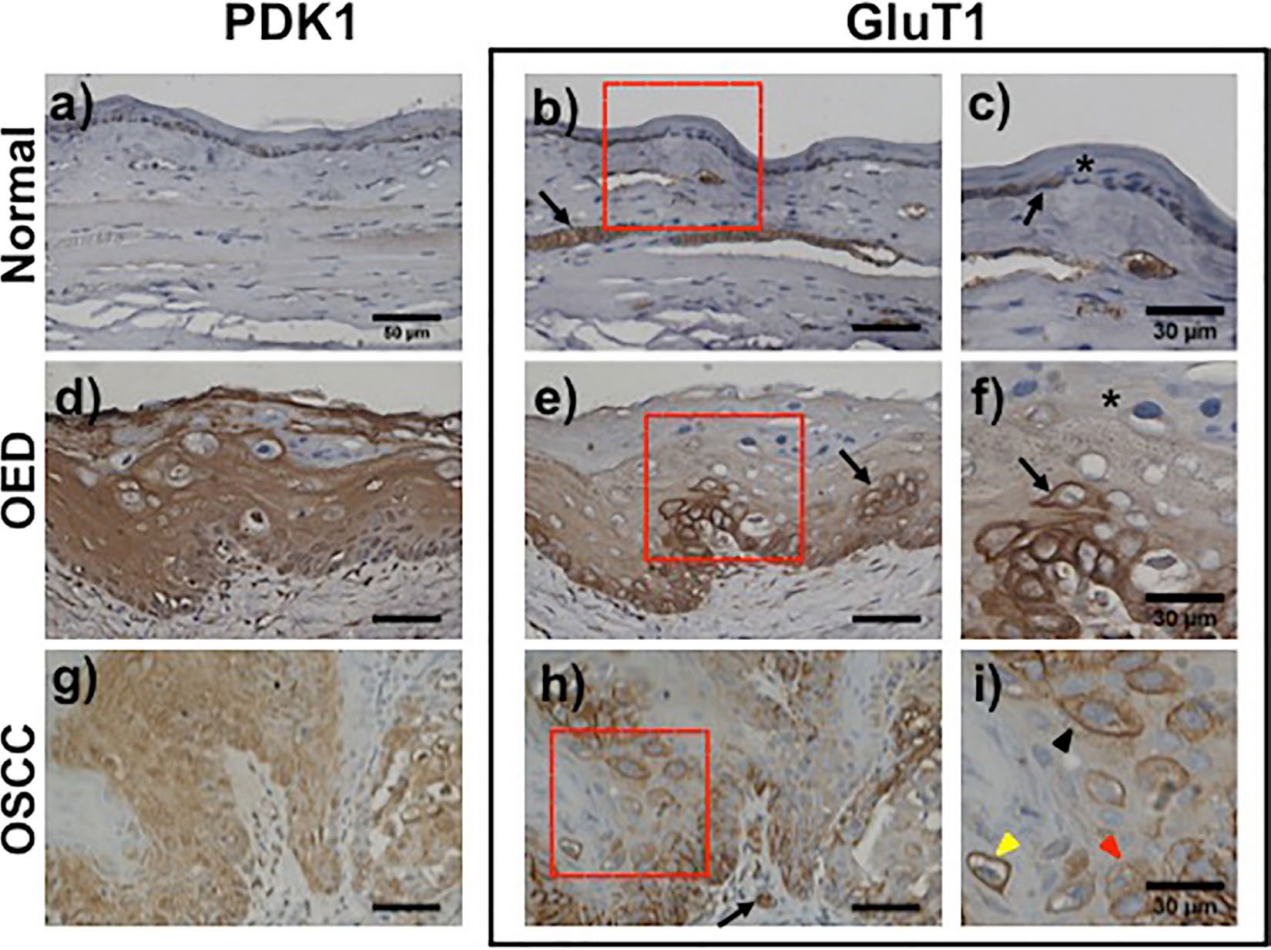
Immunohistochemistry of glycolysis markers. Immunohistochemical staining of PDK1 and GluT1 in normal (**a–c**), OED (**d**–**f**) and OSCC (**g**–**i**). Two consecutive sections were stained with PDK1 and GluT1; (**c**,**f** and **i**) are zoomed in images of GluT1 immuno staining from areas highlighted in red from normal, OED and OSCC respectively. Low expression of both PDK1 (**a**) and GluT1 (**b**) in normal tissue. GluT1 expression in muscle (**b**) is indicated by ‘→’; (**c**) mild cytoplasmic (‘→’) or no expression (‘*’) of GluT1 in normal epithelial cells; d) PDK1 staining showed a homogeneous increase in PDK1 expression in full depth of the tissue in OED; (**e**) decreasing gradient of GluT1 expression from basal cell layer to superficial keratinizing layer in OED. ‘→’ indicates an island of strong GluT1 expressing neoplastic cells; (**f**) ‘→’ and ‘*’ shows strong GluT1 expressing neoplastic cell in basal epithelium and low GluT1 expressing superficial keratinized cell respectively; (**g**) PDK1 expression in OSCC; (**h**) GluT1 expression in OSCC. GluT1 staining of red blood cells in blood vessels are indicated by “→”; (**i**) heterogeneous GluT1 expression with unique intracellular pattern of distribution in OSCC. Arrows show distribution of GluT1 highly membranous (yellow), primarily cytosolic (red) and mixed (black); Scale bar: 50 µm unless specified.

Glycolysis markers (GluT1 and PDK1) were tested to verify altered neoplastic metabolic activity. In normal epithelium, PDK1 (Fig. 6a) and GluT1 (Fig. 6b,c) exhibited cytoplasmic localization and mild membrane staining. In OED, PDK1 showed strong expression in full epithelium (Fig. 6d) while GluT1 showed strong expression in basal epipthelium that gradually decreased toward the surface (Fig. 6e). An island of strong GluT1 expressing cells is indicated (‘→’) in Fig. 6e,f shows GluT1 distribution at high magnification where surface keratinized cells showed minimal expression (‘*’) and neoplastic cell clusters in the basal layer showed strong expression (‘→’). OSCC revealed heterogeneous staining for both PDK1 (Fig. 6g) and GluT1 (Fig. 6h,i). Abnormally high PDK 1 and GluT1 expressing neoplastic cells were scattered throughout the epithelium, however GluT1 expression was more heterogeneous than PDK1. These neoplastic cells also showed heterogeneous subcellular localization of GluT1 (Fig. 6i) with membranous (yellow arrow), cytoplasmic (red arrow) or intermediate (black arrow) with both membranous and cytoplasmic localization. GluT1 expression was also evident in muscle (Fig. 6b ‘→’) and blood vessles (Fig. 6h ‘→’). PDK1 showed consistently high expression and cytoplsmic localization at all stages. IHC on a subset of areas assessed for 2-NBDG uptake showed increased GluT1 and, PDK1 expression in areas of increased 2-NBDG uptake.

### Temporal assessment of 2-NBDG fluorescence decay

**Figure 7:**
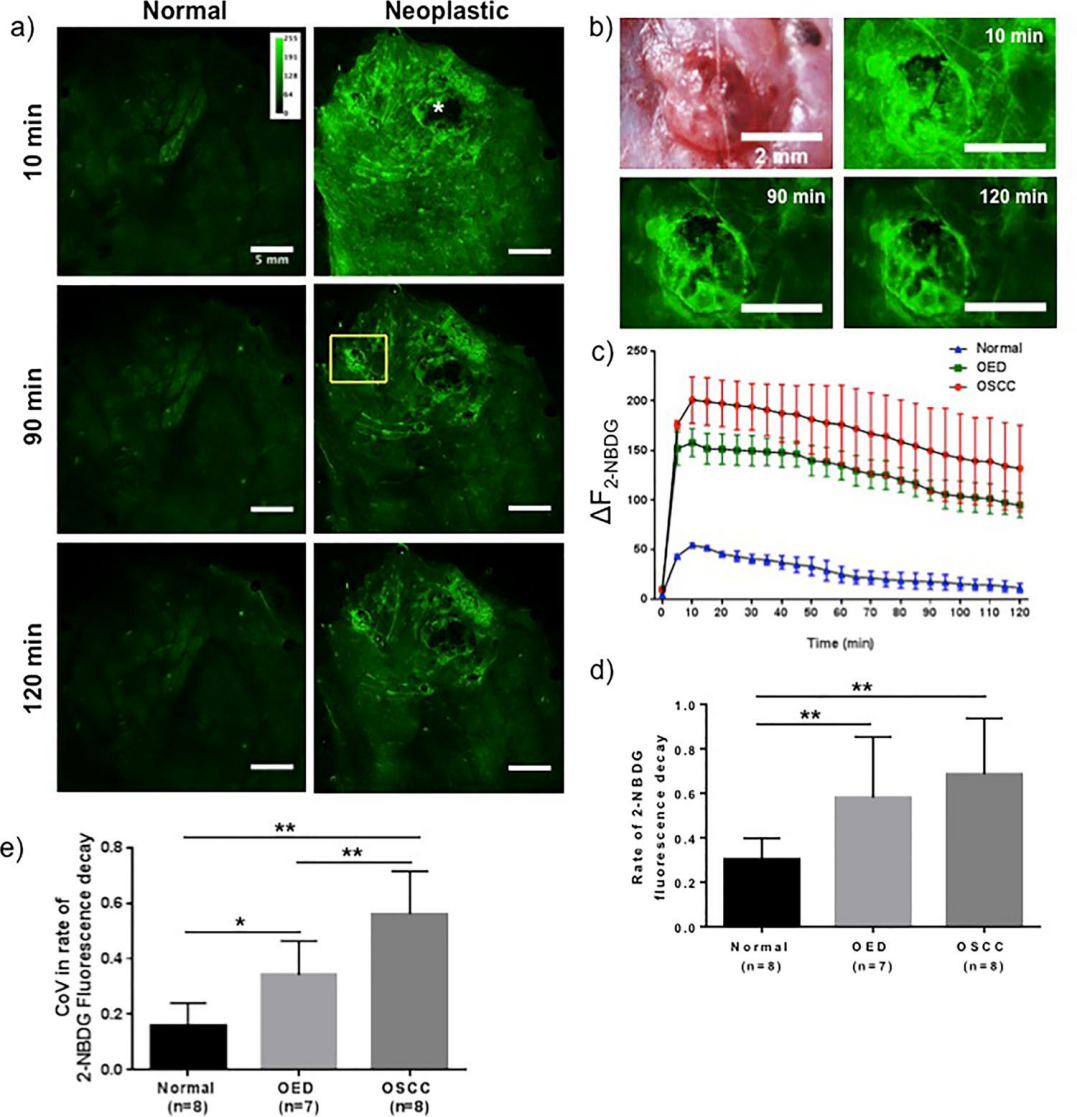
Temporal assessment of 2-NBDG fluorescence decay. (**a**) 2-NBDG fluorescence intensity over a 2-hour time period after topical application on a normal and a DMBA-treated hamster cheek pouch mucosa. Only 10 min, 90 min and 120 min time points are shown for clarity; (**b**) white light image and post 2-NBDG images of a non-necrotic tumor from the ROI (yellow outline) shown in (**a**); (**c**) change in fluorescence intensity over time with a 5 minute interval for ROIs selected from normal, OED and OSCC. Error bars represent five samplings within each site; (**d**) rate of 2-NBDG fluorescence intensity decay measured as the slope between 10 min – 120 min; (**e**) average variance in slope for normal, OED and OSCC with five samplings within each site. P-value * < 0.05, ** < 0.01.

Fig. 7 shows the temporal response of topical 2-NBDG fluorescence over a two-hour period. Normal and neoplastic tissue show a significant increase in 2-NBDG fluorescence after topical application compared to baseline autofluorescence, though this increase was significantly higher in neoplastic epithelium (Fig. 2). 2-NBDG fluorescence in normal epithelium reduced marginally over two-hours, while in neoplastic tissue fluorescence decreased rapidly after initial uptake (Fig. 7a). A large necrotic tumor (‘*’) did not show fluorescence after topical 2-NBDG. A high magnification white light image of a non-necrotic tumor (Fig. 7b), with 10, 90, and 120 minutes post 2-NBDG time-points shows temporal reduction of 2-NBDG fluorescence. Temporal fluorescence decay of normal, OED, and OSCC sites are shown in Fig. 7c. The rate of signal reduction for OSCC and OED was higher than normal (Fig. 7c), a response consistent for all specimens. 2-NBDG fluorescence decay rate was measured from the slope of a linear fitting of fluorescence intensity between 10–120 min time-points as used in similar studies^24^. Values of the slope in normal, OED, and OSCC areas in Fig. 7c are 0.39, 0.57, and 0.63, respectively, indicating faster 2-NBDG utilization of 2-NBDG by neoplastic epithelium. Figure 7d presents average slope for normal, OED, and OSCC; OED/OSCC showed a significantly increased rate of 2-NBDG fluorescence decay. To evaluate heterogeneity in glucose utilization, the coefficient of vairance (CoV) in the slope of 2-NBDG fluorescence decay was obtained from five ROIs within each assessed site (Fig. 7e). As shown in Fig. 7e, a statistically significant and increasing trend in CoV of slopes was observed from normal < OED < OSCC.

## Discussion

We investigated the potential of 2-NBDG in highlighting neoplastic regions in oral mucosa in a new *in-vivo* topical mucosal delivery approach. 2-NBDG applied directly to the fully intact mucosal surface in a living animal. This is a significant improvement to previous studies with *in-vivo* systemic injection^24,25^ or topical delivery of 2-NBDG by immersion of *ex-vivo* biopsies or resections into a solution of 2-NBDG^15,17,26,27^. Our results indicated differential responses between benign and neoplastic tissues based on 2-NBDG fluorescence intensities at single time points and temporal fluorescence decay rates. Specifically, increased intensity and decay rates were observed in neoplasia vs normal oral mucosa, which is consistent with the expectation of elevated glucose consumption (uptake and rate of utilization) in neoplasia and also with studies in which the contrast agent was delivered via injection or immersion of *ex-vivo* samples. Such studies showed enhanced tumor uptake of the probe over surrounding normal tissues^12,16,28^, measurable through increased 2-NBDG intensity.

There are distinct advantages of *in-vivo* topical mucosal delivery over systemic injection. Probe distribution via injection is usually systemic, requires high concentrations to achieve sufficient localization in the site of interest, with possible nonspecific accumulation in the liver or kidney^29,30^. In contrast, *in-vivo* topical mucosal application ensures probe delivery directly to the mucosa, making it an attractive option given uptake of the agent occurs. Our topical use of 2-NBDG was grounded in the knowledge that other sugars are taken up by the oral mucosa. D-glucose, D-fructose, and D-galactose applied directly to the oral cavity are absorbed by the mucosa using a specialized sodium ion dependent transport system^31–33^. With an internalization mechanism (glucose transporter dependent) similar to glucose^34,35^, it follows that 2-NBDG is likely to use the glucose transport system for absorption into the oral mucosa, though this remains to be investigated. As a solvent, PBS may facilitate 2-NBDG absorption; as the transport mechanism, is in part, sodium ion dependent^31,35^. Accumulation of 2-NBDG was preferential to neoplasia, similar to those of studies in which 2-NBDG was injected or applied *ex-vivo*, evident both visually and following intensity analysis^16^. 1 ml of 2-NBDG (1 mg/ml) solution was sufficient to expose the entire buccal pouch and provide contrast between normal and neoplastic tissues. This dose was lower than concentrations used in systemic injection-based studies^15,36^, with 500 µg 2-NBDG injected in mice weighing much less than hamsters. While topical delivery was straightforward, excess contrast agent on the surface had to be removed before imaging. Thus, pilot assessments were performed to establish the 30-minute topical application.

Supporting evidence of *in-vivo* 2-NBDG uptake was through *in-vivo* and *ex-vivo* tissue microscopy and cryosections for imaging 2-NBDG distribution in z-depth (Fig. 4). Figure 4 shows that epithelial 2-NBDG uptake through mucosal surface only, while previous studies with *ex-vivo* tissue biopsies may have facilitated 2-NBDG uptake by exposed submucosal cut surfaces. Significantly increased 2-NBDG fluorescence over baseline from the superficial to the basal layer following *in-vivo* topical application indicated 2-NBDG penetration into the epithelium and uptake by epithelial cells. To demonstrate that increased fluorescence was attributed to 2-NBDG we employed confocal microscopy with capabilities for spectral characterization of samples. Fluorescence spectra were consistent with 2-NBDG fluorescence emission with a distinct peak at 445–455 nm. Tissues lacking topical 2-NBDG showed very weak autofluorescence with a peak at 525 nm attributed to intracellular FAD. In all assessments 2-NBDG fluorescence was localized to epithelial cells at depths throughout the full epithelium providing strong evidence for epithelial 2-NBDG uptake through mucosal surface following topical application.

2-NBDG uptake, as indicated by hotspots of ΔF_2-NBDG_ (Fig. 2), was seen to increase substantially from normal to OED, with OSCC showing a further significant increase with the strongest uptake of 2-NBDG (Fig. 5a). Figure 2f shows a wide range of ΔF_2-NBDG_ in neoplasia, hotspots with well-defined borders, and large areas of diffuse 2-NBDG uptake consistently higher than normal. The variability in ΔF_2-NBDG_ and uptake could indicate a heterogeneous microenvironment, which in itself may present a metric for neoplasia. Increased image contrast in OSCC enabled by topical 2-NBDG could provide a distinct advantage over white light visual inspection alone, particularly in cases where lesions are not visually evident and do not protrude above the surface. While Fig. 2g,h demonstrates 2-NBDG uptake in neoplasia visible in COE, several cases showed 2-NBDG hotspots in sites not visible in COE (Fig. 3). This was evident in 6 out of 35 OEDs that were missed by COE but highlighted by 2-NBDG and later confirmed to harbor OED by histopathology. This was a significant finding as it showed the potential to identify metabolic abnormalities associated with aberrant pathology even when conventional visual detection was difficult. The animal model used allowed us to test for increased uptake of 2-NBDG with neoplasia (OED and OSCC), but represented an extreme-case scenario for tumor identification because advanced tumors were apparent on the surface. In humans, the surface generally remains flat with neoplastic growth occurring below the surface making visual identification of OSCC highly challenging by COE. Thus, we expect the benefits of 2-NBDG revealing metabolic abnormality in absence of reliable visual cues could be far greater than found in the animal model, possibly leading to a higher true positive rate, though this would have to be further investigated.

Mild dysplasia is often considered a low risk precancer and when identified in humans is frequently left untreated unless it transforms into high risk premalignancy. Thus, we considered an alternate grouping in which mild dysplasia was grouped with ‘normal/benign’. With this alternate grouping, ΔF_2-NBDG_ in moderate/severe OED remained significantly higher than normal but the difference between OED and OSCC groups became insignificant (p-value 0.12). Differences in ΔF_2-NBDG_ in other groups such as normal vs OED, normal vs OSCC and inflammation vs OSCC stayed statistically significant. We found no statistical difference between the OED subgroups, though average 2-NBDG fluorescence in mild dysplasia (27.85 ± 22.6) tended to be lower than moderate (36.51 ± 28.6) and severe dysplasia (30.42 ± 13.93). In future, assessment of OEDs with larger sample sizes should be used to reveal whether there are true differences between the OED groups. Distribution of ΔF_2-NBDG_ (Fig. 5b) indicated intensity values for inflammation overlapped between normal and OED. This resulted in the observed drop in sensitivity from 90% to 83% and specificity from 85% to 73% when inflammation was grouped with normal (Fig. 5c). Inflammation alters metabolic activity and expected to affect ΔF_2-NBDG_. Inflammation is a recognized confounding factor in COE and while 2-NBDG may delineate normal from neoplasia, inflammation presents an intermediate case. A promising result was that statistical differences were found between neoplasia and inflammation as well as controls in measures of the CoV (Fig. 7e) discussed below indicating statistically significantly higher metabolic heterogeneity in neoplasia than inflammation and could be the key to differentiating this condition from neoplasia.

The *in-vivo* temporal study on 2-NBDG utilization showed signal decay following topical application, the rates of which differed between normal and neoplasia. Presence of the fluorophore on the tissue surface and in image fields during topical application, precluded documenting 2-NBDG uptake kinetics. Interestingly, fluorescence intensity reached maximum at 10 minutes post topical application and removal of 2-NBDG (Fig. 7c), potentially indicating continued in-tissue diffusion and uptake of 2-NBDG. After reaching this maximum, 2-NBDG fluorescence decreased in all cases, indicating utilization of 2-NBDG in tissue (Fig. 7a–c). It is recognized that low levels of simultaneous uptake may still occur, but are overshadowed by utilization. Average fluorescence from OED and OSCC was consistently higher than normal throughout the 2-hour imaging. Fluorescence intensity decay, as measured by the slope between 10–120 min timepoints, primarily representing utilization of glucose, occurred faster for OED and OSCC (Fig. 7d). Therefore, topical application of 2-NBDG indicated increased uptake (higher intensities) and an increased metabolic rate (faster decays) in neoplastic epithelium. Additionally, CoV of the slope of fluorescence decay indicated significantly increased CoV within OED and OSCC sites compared to normal (Fig. 7e). CoV compares large variances in data^37^ and the results indicate a more heterogeneous metabolic microenvironment in neoplastic sites. Due to tumor heterogeneity, neoplastic sites could display different glucose consumption rates in different sub-regions. In our assessment, ROIs were analyzed using a large area WF modality encompassing a multitude of cells undergoing different metabolic reactions at various rates, is not reflective of single cell decay kinetics done in cell cultures^35,38,39^. Nonetheless, they allow us to examine the ‘averaged’ differences between normal and neoplasia and further confirm the epithelial uptake of topical 2-NBDG – without 2-NBDG uptake a differential decay response is unlikely.

Increased glucose uptake and metabolism, indicated by elevated 2-NBDG fluorescence in OSCC and OED, was validated by glucose metabolism biomarker staining (GluT1 and PDK1). GluT1 facilitates transport of glucose across the plasma membrane and allows verification of glucose uptake, which in our study is indicated by increased 2-NBDG uptake and thus increased fluorescence in neoplasia. In normal epithelium, metabolically active basal cells showed mild GluT1 expression with cytoplasmic and membranous localization, while mature superficial cells showed negligible GluT1 expression (Fig. 6c). In OED, a gradient of strong to low GluT1 expressing cells was observed from basal to superficial layers indicating highly metabolically active neoplastic cells in the basal layer (Fig. 6e,f). Interestingly, GluT1 revealed differential localization in epithelial cells in OSCC, a finding also observed in humans^40^. Islands of cells with strong GluT1 expression surrounded by areas with low expression were observed indicating a variable/heterogeneous OSCC microenvironment. Matured keratinized cells are reported to lack GluT1 expression^40^, supporting our observation of low GluT1 staining in superficial cells. In OSCC, clusters of neoplastic cells with strong GluT1 expression (Fig. 6h,i) imply a selectively higher metabolic activity in some cells, indicating a heterogeneous tumor microenvironment. Interestingly, in OSCC (Fig. 6i), we observed a mix of neoplastic cells with differing GluT1 spatial distribution, which localized either to the membrane, the cytoplasm, or a combination thereof. In humans, intracellular localization of GluT1 is associated with OSCC stage: primarily membranous in well-differentiated OSCC; membranous and cytoplasmic in poorly differentiated OSCC. We observed a similar trend in our OSCC data set. Heterogeneous GluT1 expression indicated variations in metabolic activities within neoplastic sites, which here, was demonstrated by increased CoV of the slope of 2-NBDG fluorescence decay. Beyond Glut1 expression, PDK1 expression indicates glucose utilization and metabolic activity. PDK1 participates in regulation of pyruvate oxidation, an important link between the cytoplasm and mitochondrial glycolytic pathways^41^. PDK1 allows for further downstream verification of metabolic activity between the tested conditions, indicating utilization and decay of 2-NBDG fluorescence. As opposed to GluT1, PDK1 expression was mostly cytoplasmic as glycolysis is a cytoplasmic event^42^. IHC staining of PDK1 revealed increased expression in neoplastic tissue, further associating the higher slope of fluorescence decay with downstream metabolic activity.

Changes in tissue composition and architecture, e.g. epithelial thickening and hyperkeratinization, may influence penetration depth of topical 2-NBDG. Due to 2-NBDG’s relatively low molecular weight and recent developments in mucoadhesive formulations^43,44^ such as thiolated polymers, penetration in thick tissue may improve, particularly for use in preclinical animal models. Delivery improvements can include optimization of application time, dose, and diffusion. Nonetheless, the methods outlined in this study present a viable approach to monitor metabolic alterations on the epithelial surface in preclinical studies of precancer, cancer, and even other metabolic pathologic conditions^24,45,46^.

Topical application and low doses of 2-NBDG present possibilities in clinical detection efforts in OSCC and high-risk OEDs. While 2-NBDG is currently not approved by the FDA for human use, it is a close analog to FDG used in PET imaging, and it or a similar fluorescent deoxy-glucose could potentially be advanced for human use. Such translation would require optimization in humans including careful evaluation of pharmacokinetic properties such as diffusion, active transport into cells, and clearance. Literature suggests glucose transporter mediated internalization of 2-NBDG^47^ with no *in-vivo* toxic effects^39^. WF imaging to compliment COE would provide a global view of glucose uptake and heterogeneity of glucose consumption in native environment. Thus, WF imaging with 2-NBDG adjunct to COE would improve clinical detection of neoplasia and guide areas for biopsy and histopathology. A component of successful clinical use would also entail efforts for operator/clinician training to interpret responses, such as differentiating basal level fluorescence from normal tissue and fluorescence increase in neoplastic tissue. Additionally, quantitative algorithm development and machine learning to achieve accurate automated image segmentation and disease detection could be pursued.

In conclusion, we have established feasibility of *in-vivo* optical metabolic imaging based on *in-vivo* topical mucosal delivery of a fluorescent deoxy-glucose, 2-NBDG, to oral cavity surfaces, allowing identification of neoplastic sites over a large area in a preclinical model for oral cancer and dysplasia. Results indicated *in-vivo* topical 2-NBDG highlights neoplasia associated with increased glucose uptake, metabolism, and heterogeneity in glucose consumption within the tumor microenvironment. A combination of assessments confirmed *in-vivo* uptake of 2-NBDG through the mucosal surface, with additional *in-vivo* dynamic measurements of fluorescence showing the time dependent reduction in ΔF_2-NBDG_ is much greater than in normal tissue, supporting *in-vivo* 2-NBDG utilization. These studies open several new avenues for *in-vivo* preclinical studies investigating metabolism and abnormalities of oral mucosa, with potential application to other mucosal surfaces. Additionally, the ability to perform *in-vivo* optical imaging of glucose metabolism presents new opportunities to explore the use of 2-NBDG, and other optical deoxy-glucose probes, in early epithelial neoplasia detection with optical metabolic imaging, potentially as an adjunct to COE to guide selection of areas for biopsy.

## References

[1] Foundation, T. O. C. *Information- Support- Advocacy Research… and Hope*, http://oralcancerfoundation.org/ (2017).

[2] Institute, N. C. *Cancer Stat Facts: Oral Cavity and Pharynx Cancer*, https://seer.cancer.gov/statfacts/html/oralcav.html (2017).

[3] Dost F, Le Cao K, Ford PJ, Ades C, Farah CS (2014). Malignant transformation of oral epithelial dysplasia: a real-world evaluation of histopathologic grading. Oral surgery, oral medicine, oral pathology and oral radiology.

[4] IARC. *GLOBOCAN 2012: Estimated Cancer Incidence, Mortality and Prevalence Worldwide in 2012*, http://globocan.iarc.fr/Pages/fact_sheets_population.aspx (2012).

[5] Epstein JB, Guneri P, Boyacioglu H, Abt E (2013). The limitations of the clinical oral examination in detecting dysplastic oral lesions and oral squamous cell carcinoma. Texas dental journal.

[6] Rodrigues VC, Moss SM, Tuomainen H (1998). Oral cancer in the UK: to screen or not to screen. Oral oncology.

[7] Neville BW, Day TA (2002). Oral cancer and precancerous lesions. CA: a cancer journal for clinicians.

[8] Lingen MW, Kalmar JR, Karrison T, Speight PM (2008). Critical Evaluation of Diagnostic Aids for the Detection of Oral Cancer. Oral oncology.

[9] Dang CV (2012). Links between metabolism and cancer. Genes & Development.

[10] Yuen CA, Asuthkar S, Guda MR, Tsung AJ, Velpula KK (2016). Cancer stem cell molecular reprogramming of the Warburg effect in glioblastomas: a new target gleaned from an old concept. CNS Oncology.

[11] Erdi YE (2012). Limits of Tumor Detectability in Nuclear Medicine and PET. Molecular Imaging and Radionuclide Therapy.

[12] Sheth RA, Josephson L, Mahmood U (2009). Evaluation and clinically relevant applications of a fluorescent imaging analog to fluorodeoxyglucose positron emission tomography. Journal of biomedical optics.

[13] Yokobori Y (2015). (18)F-FDG uptake on PET correlates with biological potential in early oral squamous cell carcinoma. Acta oto-laryngologica.

[14] TeSlaa T, Teitell MA (2014). Techniques to Monitor Glycolysis. Methods in enzymology.

[15] Thekkek N (2011). Pre-clinical evaluation of fluorescent deoxyglucose as a topical contrast agent for the detection of Barrett’s-associated neoplasia during confocal imaging. Technology in cancer research & treatment.

[16] Nitin N (2009). Molecular imaging of glucose uptake in oral neoplasia following topical application of fluorescently labeled deoxy-glucose. International journal of cancer.

[17] Hellebust A (2013). Vital-dye-enhanced multimodal imaging of neoplastic progression in a mouse model of oral carcinogenesis. Journal of biomedical optics.

[18] Gimenez-Conti I (1993). The hamster cheek pouch carcinogenesis model. Acta odontologica latinoamericana: AOL.

[19] Vairaktaris E (2008). The hamster model of sequential oral oncogenesis. Oral oncology.

[20] Baert JH, Veys RJ, Ampe K, De Boever JA (1996). The effect of sodium lauryl sulphate and triclosan on hamster cheek pouch mucosa. International journal of experimental pathology.

[21] Veys RJ, Baert JH, De Boever JA (1994). Histological changes in the hamster cheek pouch epithelium induced by topical application of sodium lauryl sulphate. International journal of experimental pathology.

[22] Veys RJ, Baert JH, De Boever JA (1994). Histological changes in the hamster cheek pouch epithelium induced by topical application of sodium lauryl sulphate. International journal of experimental pathology.

[23] Ahlfors EE, Dahl JE, Lyberg T (2012). The development of T cell-dominated inflammatory responses induced by sodium lauryl sulphate in mouse oral mucosa. Archives of oral biology.

[24] Rajaram N (2013). Delivery rate affects uptake of a fluorescent glucose analog in murine metastatic breast cancer. PloS one.

[25] Rajaram N, Reesor AF, Mulvey CS, Frees AE, Ramanujam N (2015). Non-invasive, simultaneous quantification of vascular oxygenation and glucose uptake in tissue. PloS one.

[26] Luo Z (2014). Widefield optical imaging of changes in uptake of glucose and tissue extracellular pH in head and neck cancer. Cancer prevention research (Philadelphia, Pa.).

[27] Lu G (2017). Detection of Head and Neck Cancer in Surgical Specimens Using Quantitative Hyperspectral Imaging. Clinical cancer research: an official journal of the American Association for Cancer Research.

[28] O’Neil RG, Wu L, Mullani N (2005). Uptake of a fluorescent deoxyglucose analog (2-NBDG) in tumor cells. Molecular imaging and biology: MIB: the official publication of the Academy of Molecular Imaging.

[29] Columbia. *Drug Absorption, Distribution and Elimination; Pharmacokinetics*, http://www.columbia.edu/itc/gsas/g9600/2004/GrazianoReadings/Drugabs.pdf (2004).

[30] Klotz, U. Pathophysiological and disease-induced changes in drug distribution volume: pharmacokinetic implications. *Clinical Pharmacokinetics* **1**, 204–218 (1976). 10.2165/00003088-197601030-00003 797498

[31] Manning AS, Evered DF (1976). The absorption of sugars from the human buccal cavity. Clinical science and molecular medicine.

[32] Oyama Y (1999). Carrier‐mediated transport systems for glucose in mucosal cells of the human oral cavity. Journal of Pharmaceutical Sciences.

[33] Rathbone MJ, Hadgraft J (1991). Absorption of drugs from the human oral cavity. International Journal of Pharmaceutics.

[34] Yamada K (2000). Measurement of glucose uptake and intracellular calcium concentration in single, living pancreatic beta-cells. The Journal of biological chemistry.

[35] Blodgett AB (2011). A fluorescence method for measurement of glucose transport in kidney cells. Diabetes technology & therapeutics.

[36] Mor A, Aizman E, George J, Kloog Y (2011). Ras inhibition induces insulin sensitivity and glucose uptake. PloS one.

[37] Reed GF, Lynn F, Meade BD (2002). Use of Coefficient of Variation in Assessing Variability of Quantitative Assays. Clinical and Diagnostic Laboratory Immunology.

[38] O’Neil RG, Wu L, Mullani N (2005). Uptake of a Fluorescent Deoxyglucose Analog (2-NBDG) in Tumor Cells. Molecular Imaging and Biology.

[39] Natarajan A, Srienc F (1999). Dynamics of Glucose Uptake by Single Escherichia coli Cells. Metabolic Engineering.

[40] Azad N (2016). Expression of GLUT-1 in oral squamous cell carcinoma in tobacco and non-tobacco users. Journal of Oral Biology and Craniofacial Research.

[41] Yu, L., Chen, X., Sun, X., Wang, L. & Chen, S. *The Glycolytic Switch in Tumors: How Many Players Are Involved*?, Vol. 8 (2017). 10.7150/jca.21125 PMC5687156 29151926

[42] Atlas, T. H. P. (SciLifeLab).

[43] Laffleur F (2014). Mucoadhesive polymers for buccal drug delivery. Drug development and industrial pharmacy.

[44] Bernkop-Schnürch A (2005). Mucoadhesive systems in oral drug delivery. Drug Discovery Today: Technologies.

[45] Marini C (2016). Discovery of a novel glucose metabolism in cancer: The role of endoplasmic reticulum beyond glycolysis and pentose phosphate shunt. Scientific Reports.

[46] Tsytsarev V (2012). *In vivo* imaging of epileptic activity using 2-NBDG, a fluorescent deoxyglucose analog. J Neurosci Methods.

[47] Speizer L, Haugland R, Kutchai H (1985). Asymmetric transport of a fluorescent glucose analogue by human erythrocytes. Biochimica et biophysica acta.

